# Assessment of the Diversity of *Pseudomonas* spp. and *Fusarium* spp. in *Radix pseudostellariae* Rhizosphere under Monoculture by Combining DGGE and Quantitative PCR

**DOI:** 10.3389/fmicb.2017.01748

**Published:** 2017-09-15

**Authors:** Jun Chen, Linkun Wu, Zhigang Xiao, Yanhong Wu, Hongmiao Wu, Xianjin Qin, Juanying Wang, Xiaoya Wei, Muhammad U. Khan, Sheng Lin, Wenxiong Lin

**Affiliations:** ^1^College of Life Sciences, Fujian Agriculture and Forestry University Fuzhou, China; ^2^Key Laboratory of Crop Ecology and Molecular Physiology, Fujian Agriculture and Forestry University Fuzhou, China; ^3^College of Crop Science, Fujian Agriculture and Forestry University Fuzhou, China; ^4^Fujian Provincial Key Laboratory of Agroecological Processing and Safety Monitoring, Fujian Agriculture and Forestry University Fuzhou, China

**Keywords:** *Radix pseudostellariae*, DGGE, *Fusarium*, *Pseudomonas*, quantitative PCR

## Abstract

*Radix pseudostellariae* is a perennial tonic medicinal plant, with high medicinal value. However, consecutive monoculture of this plant in the same field results in serious decrease in both yield and quality. In this study, a 3-year field experiment was performed to identify the inhibitory effect of growth caused by prolonged monoculture of *R. pseudostellariae*. DGGE analysis was used to explore the shifts in the structure and diversity of soil *Fusarium* and *Pseudomonas* communities along a 3-year gradient of monoculture. The results demonstrated that extended monoculture significantly boosted the diversity of *Fusarium* spp., but declined *Pseudomonas* spp. diversity. Quantitative PCR analysis showed a significant increase in *Fusarium oxysporum*, but a decline in *Pseudomonas* spp. Furthermore, abundance of antagonistic *Pseudomonas* spp. possessing antagonistic ability toward *F. oxysporum* significantly decreased in consecutively monocultured soils. Phenolic acid mixture at the same ratio as detected in soil could boost mycelial and sporular growth of pathogenic *F. oxysporum* while inhibit the growth of antagonistic *Pseudomonas sp*. CJ313. Moreover, plant bioassays showed that *Pseudomonas* sp. CJ313 had a good performance that protected *R. pseudostellariae* from infection by *F. oxysporum.* In conclusion, this study demonstrated that extended monoculture of *R. pseudostellariae* could alter the *Fusarium* and *Pseudomonas* communities in the plant rhizosphere, leading to relatively low level of antagonistic microorganisms, but with relatively high level of pathogenic microorganisms.

## Introduction

As much as 70% of medicinal plants suffer from consecutive monoculture problem, also known as replant disease or soil sickness. These problems are commonly observed in the production of many Chinese medicinal herbs, including *Radix pseudostellariae*, *Rehmannia glutinosa*, *Panax notoginseng*, etc (Zhang and Lin, 2009). *R. pseudostellariae*, a perennial tonic medicinal plant, belongs to the family Caryophyllaceae with extremely high medicinal value (Zhao W.O. et al., 2015). Consecutive monoculture of this plant in the same field leads to a serious decrease in both quality and yield of roots along with poor plant performance, which severely limited production and utilization of its medicinal plant virtues (Lin et al., 2015). Therefore, it is necessary to explore the mechanism of consecutive monoculture problems affecting the plant and develop effective control strategies for *R. pseudostellariae.*

*Fusarium* species is one of the most abundant, prevalent, and important soil fungi (Damicone and Manning, 1985). It is notorious due to the ability of attacking diversity of host plants and bring upon them diseases like vascular wilts, seedling damping off and rots of stem (Pietro et al., 2003; Punja and Parker, 2009; Chakravarty and Hwang, 2010). Similarly, the soil-borne disease caused by *F. oxysporum* in *R. pseudostellariae* fields were reported (Zhao Y.P. et al., 2015), however, the other *Fusarium* species are often overlooked. Therefore, in order to develop the full potential of the disease-suppressive microbial community in the biological control, we need more information to unravel the different roles of this potentially important species.

In recent years, more attentions were paid to develop the environment friendly and good agriculture practices for disease control. It has become important to explore the nature of microbial diversities in the soil, particularly *Pseudomonas* in different cropping periods or regime (Mendes et al., 2011). *Pseudomonas* species were reported to have a wide range of functional groups, such as plant pathogens (Samson et al., 1998), xenobiotic degraders (Clausen et al., 2002) and plant growth promoters (Patten and Glick, 2002). In addition, *Pseudomonas* species can be used as biological control agents for soil-borne pathogens, including black rot of tobacco, disease of wheat and *Fusarium* wilt (Raaijmakers and Weller, 1998; Patten and Glick, 2002; Mendes et al., 2011).

Recently, the increasing evidences suggest that plant–microbial interactions play many pivotal roles in soil quality and plant health (Lakshmanan et al., 2014; Macdonald and Singh, 2014). Li et al. (2014) reported that the peanut root exudates can selectively inhibit certain communal bacteria, such as *Gelria glutamica*, *Mitsuaria chitosanitabida*, and *Burkholderia*, but stimulate the bacterial taxon of *Desulfotomaculum ruminis* and the fungal taxa *F. oxysporum* in soil. Wu et al. (2016c) found that the amount of two pathogenic fungi (*F. oxysporum* and *Aspergillus flavus*) in the rhizosphere significantly increased after *Rehmannia glutinosa* monoculture. Wu et al. (2015) indicated that long-term continuous cropping of black pepper (*Piper nigrum* L.) could lead to a significant decrease in soil bacterial content, especially the *Pseudomonas* spp., suggesting that the soil microbes might be responsible for soil health.

Denaturing gradient gel electrophoresis (DGGE) is considered as an effective technique to directly analyze the structural and diversity of microbial communities (Kozdrój and van Elsas, 2001). The traditional method of assessing the diversity of *Fusarium* is based on enumeration and isolation of strains which were grown on selective media (Vujanovic et al., 2002). However, morphological identification of *Fusarium* species is a time-consuming and formidable task. Yergeau et al. (2005) described a PCR-DGGE method to detect the presence of multiple *Fusarium* spp. from environmental samples. The method is based on the specific amplification and separation of the transcription elongation factor-1α (Ef1α) gene. Similarly, Widmer et al. (1998) designed a primer set (PsR and PsF) which was based on the 16S rDNA gene of *Pseudomonas* spp. in 1998. When combining the PsR and PsF primers, Evans et al. (2004) developed a semi-nested PCR and DGGE to rapidly study the diversity within the genus *Pseudomonas*. Therefore, the role of soil microbial ecology in the prevention and control of plant diseases has been given more attention (Philippot et al., 2013; Cha et al., 2015). However, few studies have been carried out to understand the relationship between *Pseudomonas* and *Fusarium* of *R. pseudostellariae*, and the approaches to overcome diseases associated with this plant.

In this study, DGGE combined with qPCR technique was used to analyze the shifts of *Pseudomonas* and *Fusarium* communities in rhizosphere soil under *R. pseudostellariae* monoculture. Several microorganisms closely related to the problem of prolonged monoculture were isolated and performed for plant-microbe interactions study. Our study can help to illustrate the effects of ecological environment and root exudates on the selection of soil microbes in rhizosphere soil, and provide useful information on potential indigenous microflora for soil remediation and improvement.

## Materials and Methods

### Field Experiment

In this study, the *R. pseudostellariae* cultivar ‘Zheshen 2’ was used as the test material. The experiment was carried out at the experimental station of Fuding City, Fujian Province (27°26′ N, 120°04′ E). The experimental field which previously planted *Oryza sativa* was performed for this study with four treatments: (1) control with no *R. pseudostellariae* cultivation (CK), (2) the newly planted *R. pseudostellariae* cultivation (FP), (3) 2-year consecutive monoculture (SP), (4) 3-year consecutive monoculture (TP). The physical and chemical properties of the soil were detected before the experiment was initiated: total nitrogen of 1.83g kg^-1^, available nitrogen of 26.23 mg kg^-1^, total phosphorus of 0.47 g kg^-1^, and available phosphorus of 96.34 mg kg^-1^, total K of 8.46 g kg^-1,^ and available K of 365.21 mg kg^-1^. The station has a subtropical oceanic monsoon climate, annual mean temperature at 18.4°C. All treatments were treated with the same fertilization and field management during the experiment.

### Soil Sampling and DNA Extraction

**FIGURE 1:**
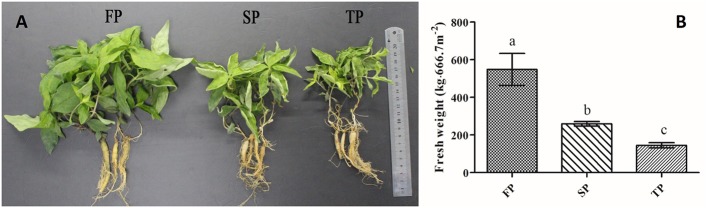
**(A)** Photographs of above and below ground components of *R. pseudostellariae* under 1-year, 2-year, and 3-year consecutive monoculture. **(B)** Yield of *R. pseudostellariae* under 1-year (FP), 2-year (SP) and 3-year consecutive monoculture (TP).

The above ground or below ground biomass of *R. pseudostellariae* become significantly different after 5 months of planting (**Figure 1A**), according to our previously study (Wu et al., 2016b). Therefore, soil samples were randomly collected from five different points at each field on April 22nd, 2015. Additionally, we harvested the plants for yield determination on July 2nd, 2015 (**Figure 1B**).

Soil samples were collected after digging the plant samples. Firstly, the loosely adhering soil was shaken off, then scraping the soil that was still attached to the root as rhizosphere soil. DNA was immediately extracted from 0.5 g soil sample per treatment using Biofast Soil Genomic DNA Extraction Kit (BioFlux, Hangzhou, China) according to the manufacturer’s protocols. We further determined the DNA concentration using Nanodrop 2000C Spectrophotometer (Thermo Scientific, United States) and then diluted it to 20 ng μL^-1^.

### PCR-DGGE and Analysis

*Fusarium*-specific PCR was performed according to the nested amplification of the Ef1α gene. The first round of PCR reactions was performed by the Ef-1 and Ef-2 primers (O’Donnell et al., 1998). It was carried out in 50 μl volumes containing 25 μl of 2 × EasyTaq PCR SuperMix (Transgen Biotech, Beijing, China), 1 μl of each primer and 40 ng template soil DNA. The program of PCR was performed by the following protocol: 95°C for 5 min, 30 cycles of denaturation (95°C for 1 min), annealing (55°C for 1 min), extension (72°C for 1 min), and 1 cycle of final extension (72°C for 10 min). The amplicons were subsequently diluted (1:20) and used for the second PCR reaction via Alfie1-GC and Alfie2 (Yergeau et al., 2005) primers. Second round PCR protocol was similar to the method of the first reaction, except for the annealing (57°C for 50 s) and extension (72°C for 50 s).

*Pseudomonas*-specific PCR was based on the nested amplification of the V6/V7 region of *Pseudomonas* spp. The first round of PCR reactions was used the PsF and PsR primers (Tan and Ji, 2010). PCR reaction was carried out in 50 μl volumes containing 25 μl of 2× EasyTaq PCR SuperMix (Transgen Biotech, Beijing, China), 1 μl of each primer and 20 ng template soil DNA. The program of PCR was performed by the following protocol: 95°C for 5 min, 30 cycles of denaturation (95°C for 1 min), annealing (64°C for 1 min), extension (72°C for 1 min), and 1 cycle of final extension (72°C for 10 min). The PCR products of first round was used to perform the second PCR reaction and the primers F968-GC1 and PsR were used (Garbeva et al., 2004). The following cycling protocol was performed for the second PCR: 1 cycle of initial denaturation at 94°C for 5 min, 10 cycle of denaturation (94°C for1 min), 1 min at 60°C (every subsequent one using a 0.5°C lower annealing temperature), and 2 min at 72°C, 1 cycle of 95°C for 5 min, 30 cycles of denaturation (95°C for 1 min), annealing (55°C for 1 min), and 1 cycle of final extension (72°C for 10 min). All PCR products were detected using 1.2% agarose gel and purified using a Gel Extraction Kit (OMEGA Bio-Tek, United States) according to the manufacturer’s instructions. The purified PCR products were used to perform DGGE experiments.

### DGGE Analysis

We performed DGGE by using an 8% (w/v) polyacrylamide gel with 35–55% and 45–60% denaturant gradients for *Fusarium*-specific and *Pseudomonas*-specific communities, respectively, using the Junyi JY-TD331A system (JUNYI, Beijing, China). DGGE was carried out at 80 V and 60°C for 12 h and 15 h in 1x TAE buffer. After electrophoresis, gels were stained with silver stain. For analysis of the molecular community profiles, gels were digitized by using the Quantity One 4.0 software (BioRad). When bands were identified, they were excised from the DGGE gel by using a sterile scalpel. After incubation overnight at 4°C, DNA was eluted from the gel. The amplicons were amplified by using the Alfie1-GC/Alfie2 and F968-GC/Psr primer sets (as mentioned before). PCR amplicons were cloned into the pEASY-T1 Cloning vector (Transgen Biotech, Beijing, China) by using manufacturer’s instructions. Sequences were compared to the sequences on GenBank of NCBI using the BlastN search method.

### Quantitative PCR for *Fusarium oxysporum* and *Pseudomonas* spp.

The fragments of *F. oxysporum* and *Pseudomonas* were cloned into the pEASY-T1 Cloning vector (TransGen Biotech Co., Beijing, China). Two plasmids were purified as described above. After determining DNA concentration, it was immediately diluted into 2, 1, 0.5, 0.1, 0.05, 0.01, 0.005, and 0.001 ng ml^-1^. The reaction of standard curve was performed following the qPCR amplification protocol as described in Supplementary Table S1. In addition, the standard curve was generated by log10 value against the threshold cycle (Ct) value.

We further performed real-time PCR quantifications of *F. oxysporum* (primer sets ITS1F and AFP308R) and *Pseudomonas* (primer sets PsF and PsR) in four soil samples, and amplification protocol as described in Supplementary Table S1. Reaction of qPCR was performed in 15 μl mixture, containing 7.5 μl TransStart Green qPCR SuperMix (Transgen Biotech, Beijing, China), 0.6 μl of each primer (10 μ M) and 20 ng DNA.

### Isolation of *Fusarium* spp.

For isolation of *Fusarium* spp., potato dextrose agar (PDA) was used to isolate and subculture the fungus. Soil suspensions were prepared by adding 10 g of fresh soil in a flask containing 90 ml of sterile water (10^-1^ g l^-1)^, 100 μl soil suspensions were plated onto PDA. Plates were incubated at 30°C for 18 h, and then each single colony was isolated and purified. *Fusarium* genomic DNA extraction was done by using CTAB-based method as described by Rogers and Bendich (1985). The primer sets ITS1F and ITS4 (Supplementary Table S2) were used for ITS amplification. PCR amplicons were sent to Shanghai BoShang for sequencing. We further used BlastN search method to compare sequences to the GenBank database.

### Isolation and Counting of *Pseudomonas* spp. with Antagonistic Activity toward *Fusarium oxysporum*

For isolation of *Pseudomonas* spp., *Pseudomonas* selective isolation agar (PSIA) (Krueger and Sheikh, 1987) was used. As described above, each soil suspensions was prepared (10^-1^ g l^-1^), after serial dilution, 60 μl soil suspensions (10^-3^ g l^-1^) were plated onto PSIA, incubated at 30°C for 30 h, and then each single colony was purified. Results were descripted as the numbers of CFU per g^-1^ (dry weight) soil.

For *in vitro* antagonism assays, we inoculated *F. oxysporum* to the center of the PDA plates and *Pseudomonas* isolates to the side of the plates at the same time. The results of antagonistic activity against *F. oxysporum* were recorded after 5 days of incubation at 30°C.

After incubation, we selected *Pseudomonas* isolates that had antagonistic activity against *F. oxysporum* for DNA extraction. *Pseudomonas* genomic DNA was extracted using the Bacteria Genomic DNA kit (CWbiotech, Beijing, China). The primer sets 27F and 1522R were used for 16S rRNA amplification. The thermal conditions are listed in the Supplementary Table S2. PCR amplicons were sent to Shanghai BoShang for sequencing. Finally, we used BlastN search method to compare sequences to the GenBank database for the identification purpose. Sequences were used Clustal X to align, and then phylogenetic trees were constructed with MEGA6.06 using a neighbor joining approach.

### Evaluation of the Pathogenicity of *Fusarium oxysporum* and Biocontrol Effects of *Pseudomonas* sp. CJ313

*Radix pseudostellariae* were planted in plastic pots and placed in a green house on December 15, 2015. The spore suspension of isolated *F. oxysporum* was added to the soil through pipette for observing the effects of *Fusarium* wilt in *R. pseudostellariae* after 5 months of planting. In order to assess biocontrol potential of *Pseudomonas* spp., the effect of isolated strain CJ313 was examined after 15 days of its exogenous addition. We added equal amount of LB as a control (CK) at the same time. Each treatment has three replicates. After 16 days, we collected rhizospheric soil from two treatments, then soil samples were immediately used to extract DNA and qPCR of *F. oxysporum* and *Pseudomonas* spp. as described above.

### The Effect of Phenolic Acids on the Growth of Isolated *Fusarium oxysporum*, *Pseudomonas* sp. CJ313 and *Pseudomonas* sp. CJ361

Based on our previous HPLC results of phenolic acids in the *R. pseudostellariae* rhizosphere (Wu et al., 2016a), we prepared the solutions of eight phenolic acids (*p*-hydroxybenzoic acid, gallic acid, coumaric acid, syringic acid, vanillic acid, ferulic acid, vanillin and benzoic acid) and their mixture to assess its effect on the growth of isolated *F. oxysporum.* The ratio of their mixtures was the same as detected in the soil. We prepared the 10-fold dilution of soil extract agar medium (SEM), and added the phenolic acids into the SEM to reach final concentrations 30, 60, 120, 240, 480, 960 μ mol L^-1^. We inoculated isolated *F. oxysporum* onto the SEM plates to assess the mycelium growth mediated by phenolic acids. There were three replicates for each treatment. After incubation at 28°C for 8 days, we recorded the mycelium diameter. Likewise, isolated *F. oxysporum* was inoculated into 10-fold dilution of SEM by adding the phenolic acid mixtures, and solution was incubated at 200 rpm and 30°C for 7 days. *F. oxysporum* spores were counted by a hemocytometer.

We also detected the effects of eight phenolic acids and their mixtures on the growth of isolated *Pseudomonas* sp. CJ313 and CJ361. Specifically, the isolated *Pseudomonas* sp. CJ313 and CJ361 were determined by adding the phenolic acids to a LB medium with 8-fold dilution. After 8–10 h incubation at 200 rpm and 30°C, we determined the bacterial density at 600 nm using a microplate reader (Thermo Scientific Multiskan MK3, Shanghai, China).

### Statistical Analyses

For all parameters, multiple comparison was carried out by one-way analysis of variance (ANOVA) followed by LSD’s test (*P* ≤ 0.05) using DPS 7.05 software. PCA analysis was performed by SPSS 20.0 software. DGGE for detecting the band was performed with the Quantity one v4.6.2 software.

## Results

### The Morphology and Yield of *R. pseudostellariae* under Consecutive Monoculture

We observed that plants of FP displayed more aboveground biomass and less adventitious roots relative to continuously monocultured plants of SP and TP (**Figure 1A**). Moreover, our results revealed that the yield of newly planted *R. pseudostellariae* roots (FP) was significantly (*P* ≤ 0.05) higher that of 2-year consecutive monoculture (SP) and 3-year consecutive monoculture (TP) (**Figure 1B**).

### *Fusarium*-Specific DGGE

**FIGURE 2:**
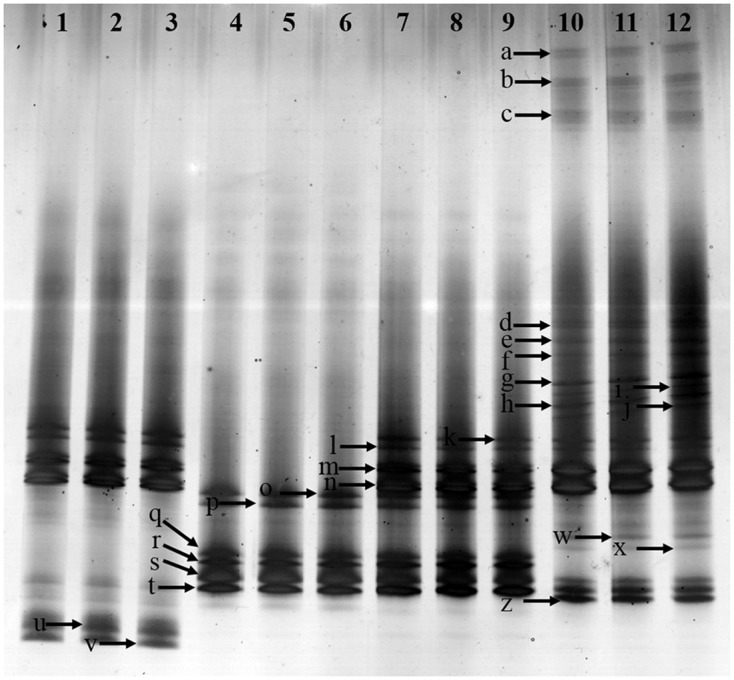
Denaturing gradient gel electrophoresis (DGGE) gel showing community structures of *Fusarium* elongation factor genotypes (EF1α) amplified from soil extracted DNA. Lanes 1, 2 and 3-control with no *Radix pseudostellariae* cultivation (CK), lanes 4, 5 and 6- the newly planted *R. pseudostellariae* cultivation(FP), lanes 7, 8, and 9- 2-year consecutive monoculture(SP), lanes 10, 11, and 12- 3-year consecutive monoculture (TP). Band positions are marked with an arrow and correspond to data in **Table 2**.

**FIGURE 3:**
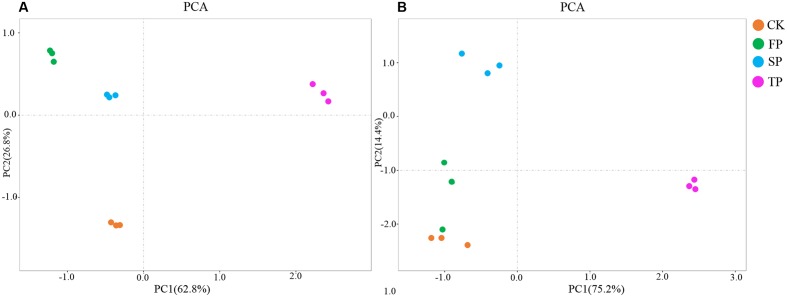
Principal component analysis of *Fusarium* DGGE results **(A)** *Pseudomonas* DGGE results **(B)**. CK, control with no *R. pseudostellariae* cultivation. FP, the newly planted *Radix. pseudostellariae* cultivation. SP, 2-year consecutive monoculture. TP, 3-year consecutive monoculture.

*Fusarium*-specific PCR-DGGE analysis showed that the rhizosphere *Fusarium* community structures changed with the increasing years of monoculture (**Figure 2**). The principal component analysis (PCA) of the DGGE profile was performed to demonstrate the relative position of four soil samples. In PCA, first principal component explained 57.70% of variance and second principal component 28.44% of total variance (**Figure 3A**). Furthermore, PCA showed that the *Fusarium* community in CK, FP and SP was separated from TP by the first principal component, and FP was separated from SP by the second principal component (**Figure 3A**).

The diversity of *Fusarium*-specific DGGE was also determined. The study revealed that Simpson, Shannon, evenness and Brillouin’s index of *Fusarium* communities significantly increased with prolonged or increasing years of monoculture (*P* ≤ 0.05) (**Table 1**).

**Table 1:** Estimated Simpson, Shannon, Evenness and Brillouin’s indices for all the samples using *Fusarium*-specific DGGE.

Treatments	Simpson	Shannon	Evenness	Brillouin
CK	0.8632 ± 0.0041c	2.9324 ± 0.0218c	2.9324 ± 0.0218c	2.9011 + 0.0216c
FP	0.8497 ± 0.002d	2.762 ± 0.0099d	2.762 ± 0.0099d	2.7279 + 0.0097d
SP	0.8801 ± 0.0016b	3.1098 ± 0.013b	3.1098 ± 0.013b	3.0784 + 0.0146b
TP	0.9364 ± 0.0018a	4.0264 ± 0.0199a	4.0264 ± 0.0199a	3.9928 ± 0.0188a


*Different letters within a column indicate significant differences according to LSD (*P* ≤ 0.05). CK, control with no *Radix pseudostellariae* cultivation. FP, the newly planted *R. pseudostellariae* cultivation. SP, 2-year consecutive monoculture. TP, 3-year consecutive monoculture).*

### Analysis of the DGGE Bands of *Fusarium* spp.

In order to further extract more detailed information from the DGGE bands in this study, excised bands from DGGE were sequenced. A total of 17 bands were identified in rhizospheric soil (**Table 2**). The *Fusarium* spp. belonged to 5 species, e.g., *F. oxysproum* (band a, b, c, g, h, k, l, m and n), *F. solani* (band p, q, r, s and t), *F. asiaticum* (band d), *F. falciforme* (band e), *F. foetens* (band f). Specifically, the bands of *F. oxysporum* significantly increased along with years of continuous cropping.

**Table 2:** Sequencing of the identified bands in the *Fusarium*–specific DGGE gel.

Bands	Species	Identity	NCBI accession numbers
a	*F. oxysporum*	99%	KT224070.1
b	*F. oxysporum*	94%	KT224065.1
c	*F. oxysporum*	96%	KF728239.1
g	*F. oxysporum*	99%	LC177322.1
h	*F. oxysporum*	99%	KT224113.1
k	*F. oxysporum*	99%	AB674272.1
l	*F. oxysporum*	99%	AB674271.1
m	*F. oxysporum*	97%	JF430176.1
n	*F. oxysporum*	99%	AB674272.1
p	*F. solani*	99%	KU361412.1
q	*F. solani*	99%	KT224163.1
r	*F. solani*	99%	KP267291.1
s	*F. solani*	99%	KR108757.1
t	*F. solani*	99%	KU361425.1
d	*F. falciforme*	99%	LC177299.1
e	*F. foetens*	95%	JX298790.1
f	*F. asiaticum*	99%	KY283868.1


*Note: Identification letters refer to the identified bands in **Figure 2**.*

### *Pseudomonas*-Specific DGGE

**FIGURE 4:**
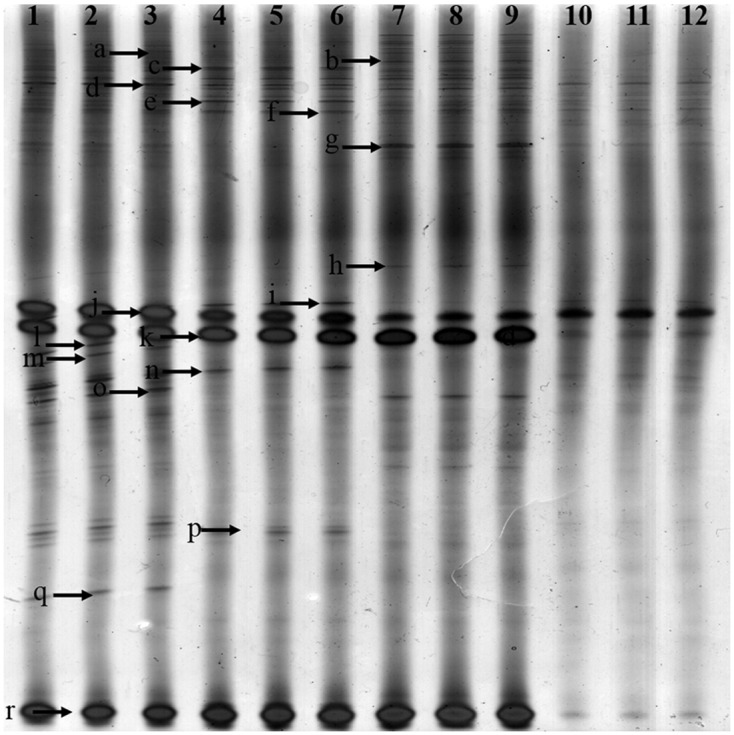
Denaturing gradient gel electrophoresis gel showing community structures of *Pseudomonas* amplified from soil extracted DNA. Lanes 1, 2, and 3-control with no *Radix pseudostellariae* cultivation (CK), lanes 4, 5, and 6- the newly planted *R. pseudostellariae* cultivation(FP), lanes 7, 8, and 9- 2-year consecutive monoculture(SP), lanes 10, 11, and 12- 3-year consecutive monoculture(TP). Band positions are marked with an arrow and correspond to data in **Table 4**.

*Pseudomonas*-specific PCR-DGGE analyses showed significantly changed *Pseudomonas* community structures in the rhizosphere with increasing years of monoculture (**Figure 4**). Likewise, we performed PCA to demonstrate the relative position of four soil samples. In PCA, the first principal component explained 83.30% of variance and second principal component 9.0% of total variance (**Figure 3B**). Furthermore, PCA showed the *Pseudomonas* community in CK, FP and SP were separated from the microbial community in TP by principal component 1, and the community in FP and CK was separated from the microbial communities in SP and TP by principle component 2 (**Figure 3B**).

The diversity of visible bands, Shannon and Brillouin’ index of *Pseudomonas* community significantly decreased with increasing years of monoculture (*P* ≤ 0.05). However, the opposite was true for the Simpson’ index of the *Pseudomonas* community. There was no significant difference in evenness index among the four samples (**Table 3**).

**Table 3:** Estimated Simpson, Shannon, Evenness and Brillouin’s indices for all the samples using *Pseudomonas*-specific DGGE.

Treatments	Simpson	Shannon	Evenness	Brillouin
CK	0.9394 ± 0.0036b	3.4111 ± 0.0373a	0.7473 ± 0.0057a	2.0966 ± 0.131a
FP	0.9018 ± 0.0087c	2.8775 ± 0.0376c	0.7365 ± 0.0096a	1.707 ± 0.0874b
SP	0.932 ± 0.0131bc	3.1293 ± 0.0467b	0.7367 ± 0.011a	1.8585 ± 0.0217b
TP	1.1522 ± 0.0303a	2.6477 ± 0.0278d	0.7385 ± 0.0078a	0.5653 ± 0.0914c


*Different letters within a column indicate significant differences according to LSD (*P* ≤ 0.05). CK, control with no *Radix pseudostellariae* cultivation. FP, the newly planted *R. pseudostellariae* cultivation. SP, 2-year consecutive monoculture. TP, 3-year consecutive monoculture).*

### Analysis of the DGGE Bands of *Pseudomonas* spp.

To further extract more detailed information from the DGGE bands, we excised and sequenced bands from DGGE. A total of 15 bands were identified in rhizospheric soil (**Table 4**). *Pseudomonas* spp. could be further divided into five species, e.g., *Pseudomonas lutea* (band c and k), *Pseudomonas fluorescens* (band d), *Pseudomonas aeruginosa* (band l), *Pseudomonas knackmussii* (band o and r), *Pseudomonas* sp. (band j) and uncultured bacterium (a, b, f, g, i, m, p and q).

**Table 4:** Sequencing of the identified bands in the *Pseudomonas* –specific DGGE gel.

Bands	Species	Identity	NCBI accession numbers
a	Uncultured bacterium	96%	KM318828.1
b	Uncultured bacterium	98%	EU408015.1
c	*Pseudomonas lutea*	99%	NZ_JRMB010000003.1
d	*Pseudomonas fluorescens*	95%	KX503978.1
e	Uncultured bacterium	94%	JQ054733.1
f	Uncultured bacterium	94%	KM318828.1
g	Uncultured bacterium	97%	KR309072.1
i	Uncultured bacterium	97%	DQ468062.1
j	*Pseudomonas* spp.	88%	AY365077.1
k	*Pseudomonas lutea*	99%	NZ_JRMB010000004.1
l	*Pseudomonas aeruginosa*	93%	AB793685.1
m	Uncultured bacterium	93%	JQ054733.1
o	*Pseudomonas knackmussii*	93%	NZ_GH322950.1
p	Uncultured bacterium	93%	DQ129211.2
q	Uncultured bacterium	93%	KM323906.1
r	*Pseudomonas knackmussii*	82%	NZ_GH322950.1


### Abundance of *Pseudomonas* and *Fusarium oxysporum* by Quantitative PCR

**FIGURE 5:**
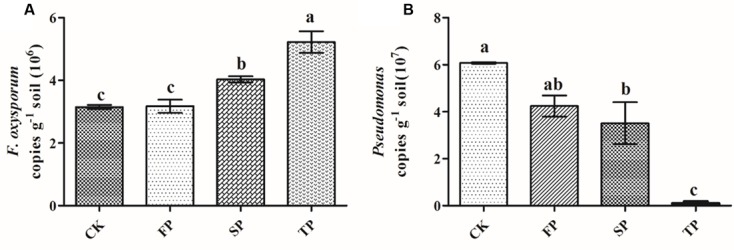
Quantification of *Fusarium oxysporum* **(A)** and *Pseudomonas* **(B)** in control (CK), the newly planted *Radix pseudostellariae* cultivation (FP), 2-year consecutive monoculture (SP), and 3-year consecutive monoculture (TP) by quantitative PCR. Data are means ± standard errors (one-way analysis of variance, *n* = 4).

First, standard curves of y = -0.2487x + 9.898 (*R*^2^ = 0.997) and y = -0.271x + 9.8309 (*R*^2^ = 0.990) were developed for *Pseudomonas* and *F. oxysporum* qPCR analyses respectively. The amount of *F. oxysporum* was significantly (*P* ≤ 0.05) higher in continuous monoculture soils (SP and TP) than in control (CK) and the newly planted soils (FP) (**Figure 5A**). The result of qPCR was consistent with the *Fusarium*-specific DGGE results (**Table 1**). However, the opposite was true for the qPCR result of *Pseudomonas* (**Figure 5B**).

### Isolation and Screening for *F. oxysporum* with High Pathogenicity

In our study, we separated and sequenced one strain of *F. oxysporum.* We found that the isolated *F. oxysporum* quickly led to wilt disease on the tissue culture of *R. pseudostellariae* (**Figure 7A**), and it also occurred in pots with *F. oxysporum* (**Figure 7B**). These results demonstrated that isolated *F. oxysporum* had the high pathogenicity on *R. pseudostellariae*.

### Screening for *Pseudomonas* Isolates with Antagonistic Activity toward *F. oxysporum*

**FIGURE 6:**
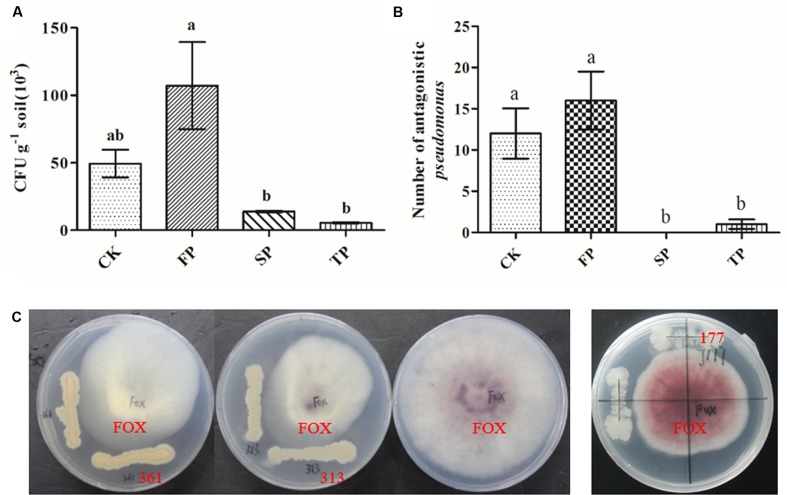
*Pseudomonas* populations (CFU g^-1^ of dry soil) in rhizosphere soils under four different samples **(A)**, Number of *Pseudomonas* spp. with antagonistic activity against *F. oxysporum* in four different samples **(B)**. Petri plates used for evaluation of antagonistic activity of *Pseudomonas* strains 313, 361 and 117 against *F. oxysporum* **(C)**. CK, FP, SP and TP represent the control, newly planted, 2-year, and 3-year consecutively monoculture soils, respectively. Data are means ± standard errors (one-way analysis of variance, *n* = 3).

For *in vitro* antagonism assays, we screened a total of 317 *Pseudomonas* isolates from four different soils. The results showed that the isolation frequencies of *Pseudomonas* were significantly higher in FP than SP and TP. The highest isolation frequencies were found in the newly planted (FP) soil (**Figure 6A**). *In vitro* antagonism assays, the number of *Pseudomonas* spp. with antagonistic activity toward *F. oxysporum* significantly declined with prolonged monoculture (**Figure 6B**). These isolation frequencies were similar to results of *Pseudomonas* obtained by qPCR. Approximately 17.4% (87 of 317) of all isolates showed the antagonistic activity. Strain 313 and 361 but not 117 had antagonistic activity against *F. oxysporum* (**Figure 6C**). The sequences of *Pseudomonas* sp. CJ313 and *Pseudomonas* sp. CJ361 isolates were obtained to perform phylogenetic tree analysis. The neighbor-joining method generated a dendrogram with two main branches, where the first branch included *Pseudomonas* sp. CJ361 and the second branch comprised *Pseudomonas* sp. CJ313 (Supplementary Figure S1).

### Biocontrol Effects of *Pseudomonas* sp. CJ313

**FIGURE 7:**
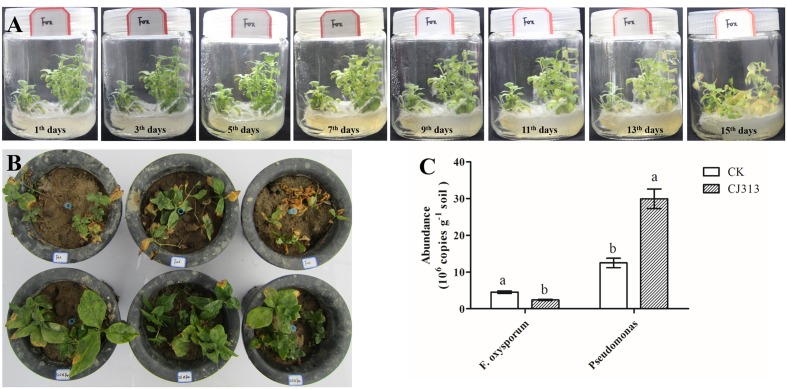
Assessment of the pathogenic potential of isolated *F. oxysporum* **(A)**, the biocontrol potential of *Pseudomonas* sp. CJ313 against *F. oxysporum* (FOX) **(B)**, Quantification of *F. oxysporum* and *Pseudomonas* spp. from two samples. Data are means ± standard errors (one-way analysis of variance, *n* = 4).

We further evaluated the antagonism of *Pseudomonas* CJ313 to *F. oxysporum*. In the pot experiment, we found that the isolated *Pseudomonas* CJ313 significantly inhibited the growth of *F. oxysporum*, and the *R. pseudostellariae* grew well without disease symptoms during the period of experiment (**Figure 7B**). Moreover, qPCR indicated that the abundance of *Pseudomonas* was significantly higher in *Pseudomonas*. CJ313 treatment than in control (CK), whereas *F. oxysporum* showed the opposite trend (**Figure 7C**). The results clearly showed that strain *Pseudomonas*. CJ313 has the potential of biological control. The results further suggested that exogenous antagonism of *Pseudomonas* could be effective against *F. oxysporum* infection. In addition, the results also demonstrated that the imbalance of these two strains (*Pseudomonas* sp. CJ313 and *F. oxysporum*) could be an important cause of the continuous cropping related diseases.

### The Effect of Phenolic Acids on the Growth of Isolated *Fusarium oxysporum*, *Pseudomonas* spp.

**FIGURE 8:**
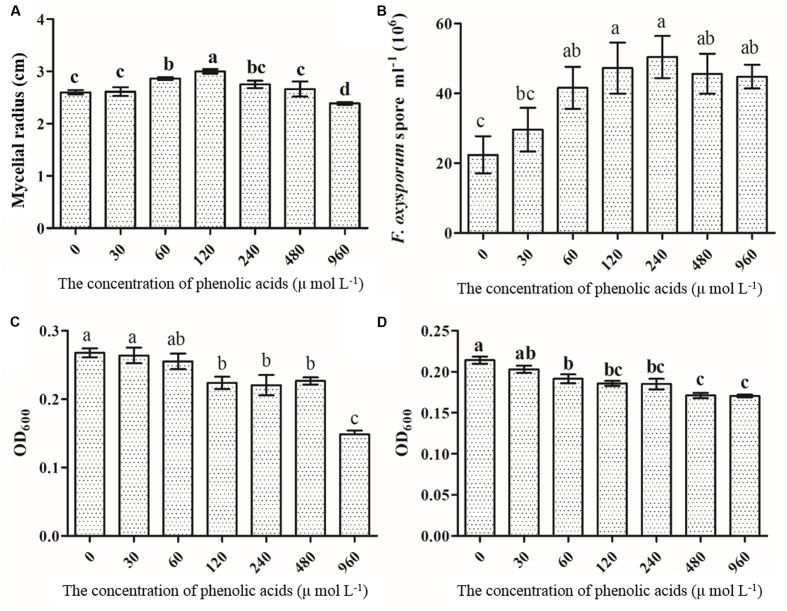
The effects of phenolic acid mixture on the growth of *F. oxysporum* **(A)**, sporulation of *F. oxysporum* **(B)**, *Pseudomonas* sp. CJ313 **(C)**, *Pseudomonas* sp. CJ361 **(D)**. The proportion of phenolic acids was the same as the ratio detected in the rhizosphere soil of *Radix pseudostellariae*. CK, FP, SP and TP represent the control, newly planted, 2-year, and 3-year consecutively monoculture soils, respectively. Data are means ± standard errors (one-way analysis of variance, *n* = 4).

The results showed that mycelial and sporular growth of *F. oxysporum* was significantly promoted by phenolic acid mixture (**Figures 8A,B**). Further analysis showed that *p*-hydroxybenzoic acid, vanillin, coumaric acid and ferulic acid could significantly promoted mycelial growth of *F. oxysporum* among the eight phenolic compounds (Supplementary Figure S2). The results also indicated that the growth promotion by mixture was more than that of single phenolic acid on *F. oxysporum* (**Figure 8A**). However, the mixture significantly inhibited *Pseudomonas* sp. CJ313 (**Figure 8C**) and *Pseudomonas* sp. CJ361 (**Figure 8D**) growth. Among them, vanillic acid and syringic acid has the more inhibitory effects on *Pseudomonas* sp. CJ313 than others (Supplementary Figure S3). Likewise, coumaric acid, ferulic acid syringic acid had the greatest inhibitory effect on *Pseudomonas* sp. CJ361 (Supplementary Figure S4). The results indicated that certain allelochemicals of *R. pseudostellariae* root exudates possessed the selective effects on rhizosphere microbes.

## Discussion

Our studies presented a significant decline in the yield of *R. pseudostellariae* along with less aboveground biomass in consecutive monoculture field (**Figure 1**). Recently, researchers have focused on the biological relationships between plants and rhizosphere microorganisms, which are essential for plant growth and health (Haney and Ausubel, 2015; Lebeis et al., 2015). The study of *F. oxysporum* has become common due to its ability to cause diseases of important economic crops (Gordon et al., 1989; Gordon and Martyn, 1997). DGGE results revealed significant changes in *Pseudomonas* and *Fusarium* communities in the rhizosphere of *R. pseudostellariae* with prolonged monoculture (**Figures 2**, **4**). Based on the DGGE analysis of *Fusarium*, we indicated that prolonged monoculture of *R. pseudostellariae* led to a significant increase in *Fusarium* species, especially *F. oxysporum* (**Table 1**). Quantitative PCR assay confirmed the increase in *F. oxysporum* with the increasing years of monoculture (**Figure 3**). These results are supported by the work of different researchers as stated that *F. oxysporum* is one of main pathogenic species to plants under monoculture regime (Wu et al., 2016b,c).

Due to an extensive distribution of *Pseudomonas* species in the environment, several studies reported an abundance of antagonistic *Pseudomonas* species, which controls specialized pathogens that are responsible for disease suppression in soils (Gorlach-Lira and Stefaniak, 2009; Mendes et al., 2011). Our study of *Pseudomonas*-DGGE revealed that the diversity of *Pseudomonas* spp. significantly declined with the prolonged monoculture. More importantly, it was found that the relative abundances of antagonistic *Pseudomonas* spp. declined in soils under consecutive monoculture, and a similar tendency was recorded for other *Pseudomonas* species studied in the selective medium assay. Similar effects of plants on the abundance of antagonistic *Pseudomonas* spp. under monoculture were found by Gorlach-Lira and Stefaniak (2009). Hence, the abundance of the *Pseudomonas* populations in soil of *R. pseudostellariae* were seriously affected by monoculture. In addition, it was also found that the abundance of *Pseudomonas* spp. having antagonistic activities against *F. oxysporum* significantly decreased with the increasing years of monoculture, and this was confirmed by the *in vitro* antagonism assays. This important antagonistic interaction effects between *Pseudomonas* and *F. oxysporum* need particular attention in disease management under a clear cropping system of *R. pseudostellariae*. Therefore, it is necessary to make robust inferences about balance between *Pseudomonas* communities and *Fusarium* of *R. pseudostellariae*.

Our previous study revealed that most phenolic acids of *R. pseudostellariae* from rhizosphere soil indicated no direct autotoxicity toward tissue culture seedlings of *R. pseudostellariae* (Wu et al., 2016a). Besides, many researchers did not support the assumption that the concentrations of allelochemicals in the soil were sufficient to directly influence the development of host plants or neighboring plants (Ehlers, 2011; Weidenhamer et al., 2013). A growing number of researchers reported that the microflora disorder mediated by plant root exudates was the crucial factor leading to plant consecutive monoculture problems (Wu et al., 2014). Root exudates have selective effects on certain microorganisms in the soil and can promote or inhibit the growth of a certain population (Haichar et al., 2008; Hartmann et al., 2009). In this study, the results indicated that the phenolic acid mixture had a significant improvement on the growth of mycelial and spore of pathogenic *F. oxysporum* (**Figures 8A,B**). However, phenolic acid mixture could greatly inhibit the growth of antagonistic *Pseudomonas* sp. CJ313 and CJ361 (**Figures 8C,D**). Zhou and Wu (2012) observed that the abundance of *F. oxysporum* in soil was significantly increased by *p*-coumaric acid, which led to the severity of *Fusarium* wilt in field conditions. Wu et al. (2016a) reported that phenolic acid, such as syringic acid, significantly promoted the growth of *Talaromyces helicus* and *Kosakonia sacchari*, and inhibited growth of *Bacillus pumilus*. Bais et al. (2002) found that rosmarinic acid had a significant and deleterious effect on *Pseudomonas aeruginosa*. Furthermore, plant bioassays with representative isolates of *Pseudomonas* showed that *Pseudomonas* CJ313 had a good performance that protected *R. pseudostellariae* from infection by *F. oxysporum* (**Figure 7B**). Combined with above-mentioned results, we can draw robust inferences that the imbalance of belowground microbial community resulted in the poor growth of monocultured *R. pseudostellariae* by root exudates.

## Conclusion

Based on multifaceted approaches, such as cultural-independent and culture-dependent analyses, this study indicated that *R. pseudostellariae* biomass decreased under 3-year extended monoculture resulted from two important factors: (i) the decrease of antagonistic microorganisms (*Pseudomonas* sp. CJ313 and CJ361) against pathogens (*F. oxysporum*) might be due to selective inhibitory effect of root exudates, especially phenolic compounds and (ii) an increase of *F. oxysporum* which significantly induced the poor growth of *R. pseudostellariae* at a time when pathogenic microbes (*F. oxysporum*) have become dominant. (iii) Isolated *Pseudomonas* CJ313 of its exogenous addition could protected *R. pseudostellariae* from infection by *F. oxysporum*. These results are very important in the development of potential management approaches to solve the *R. pseudostellariae* problems under consecutive monoculture. However, additional works are still needed to explore the relationship between aboveground plant performance and belowground microbial diversity.

## Author Contributions

WL and JC conceived the study; JC wrote the paper; JC, and LW performed experiments; JC, SL, and ZX performed the statistical analyses; HW, XQ, YW, XW, and JW were involved in field management. MK assisted in English correction. All authors discussed the results and commented on the manuscript.

## Conflict of Interest Statement

The authors declare that the research was conducted in the absence of any commercial or financial relationships that could be construed as a potential conflict of interest.

## References

[B1] Bais H. P. Walker T. S. Schweizer H. P. Vivanco J. M. (2002). Root specific elicitation and antimicrobial activity of rosmarinic acid in hairy root cultures of *Ocimum basilicum*. *Plant Physiol. Biochem.* 40 983–995. 10.1016/s0981-9428(02)01460-2

[B2] Cha J. Y. Han S. Hong H. J. Cho H. Kim D. Kwon Y. (2015). Microbial and biochemical basis of a *Fusarium* wilt-suppressive soil. *ISME J.* 10 119–129. 10.1038/ismej.2015.95 26057845 PMC4681868

[B3] Chakravarty P. Hwang S. F. (2010). Effect of an ectomycorrhizal fungus, *Laccaria laccata*, on *Fusarium* damping-off in *Pinus banksiana* seedlings. *For. Pathol.* 21 97–106. 10.1111/j.1439-0329.1991.tb00949.x

[B4] Clausen G. B. Larsen L. Johnsen K. Julia R. D. L. Aamand J. (2002). Quantification of the atrazine-degrading *Pseudomonas* sp. strain ADP in aquifer sediment by quantitative competitive polymerase chain reaction. *FEMS Microbiol. Ecol.* 41 221–229. 10.1111/j.1574-6941.2002.tb00983.x 19709256

[B5] Damicone J. P. Manning W. J. (1985). Frequency and pathogenicity of *Fusarium* spp. isolated from first-year asparagus grown from transplants. *Plant Dis.* 69 413–416.

[B6] Ehlers B. K. (2011). Soil microorganisms alleviate the allelochemical effects of a thyme monoterpene on the performance of an associated grass species. *PLOS ONE* 6:e26321 10.1371/journal.pone.0026321 PMC3219634 22125596

[B7] Evans F. F. Seldin L. Sebastian G. V. Kjelleberg S. Holmström C. Rosado A. S. (2004). Influence of petroleum contamination and biostimulation treatment on the diversity of *Pseudomonas* spp. in soil microcosms as evaluated by 16S rRNA based-PCR and DGGE. *Lett. Appl. Microbiol.* 38 93–98. 10.1111/j.1472-765x.2003.01455.x 14746538

[B8] Garbeva P. Veen J. A. Elsas J. D. (2004). Assessment of the diversity, and antagonism towards *Rhizoctonia solani* AG3, of *Pseudomonas* species in soil from different agricultural regimes. *FEMS Microbiol. Ecol.* 47 51–64. 10.1016/s0168-6496(03)00234-4 19712346

[B9] Gordon T. R. Martyn R. D. (1997). The evolutionary biology of *Fusarium oxysporum*. *Annu. Rev. Phytopathol.* 35 111–128. 10.1146/annurev.phyto.35.1.111 15012517

[B10] Gordon T. R. Okamoto D. Jacobson D. J. (1989). Colonization of muskmelon and nonsusceptible crops by *Fusarium oxysporum* f. sp. *melonis* and other species of *Fusarium*. *Phytopathology* 79 1095–1100. 10.1094/phyto-79-1095

[B11] Gorlach-Lira K. Stefaniak O. (2009). Antagonistic activity of bacteria isolated from crops cultivated in a rotation system and a monoculture against *Pythium debaryanum* and *Fusarium oxysporum*. *Folia Microbiol.* 54 447–450. 10.1007/s12223-009-0062-1 19937218

[B12] Haichar F. Z. Marol C. Berge O. Rangel-Castro J. I. Prosser J. I. Balesdent J. (2008). Plant host habitat and root exudates shape soil bacterial community structure. *ISME J.* 2 1221–1230. 10.1038/ismej.2008.80 18754043

[B13] Haney C. H. Ausubel F. M. (2015). Plant microbiome blueprints. *Science* 349 788–789. 10.1126/science.aad0092 26293938

[B14] Hartmann A. Schmid M. Tuinen D. V. Berg G. (2009). Plant-driven selection of microbes. *Plant Soil* 321 235–257. 10.1007/s11104-008-9814-y

[B15] Kozdrój J. van Elsas J. D. (2001). Structural diversity of microorganisms in chemically perturbed soil assessed by molecular and cytochemical approaches. *J. Microbiol. Methods* 43 197–212. 10.1016/s0167-7012(00)00197-4 11118654

[B16] Krueger C. L. Sheikh W. (1987). A new selective medium for isolating *Pseudomonas* spp. from water. *Appl. Environ. Microbiol.* 53 895–897. 3579287 10.1128/aem.53.4.895-897.1987 PMC203776

[B17] Lakshmanan V. Selvaraj G. Bais H. P. (2014). Functional soil microbiome: belowground solutions to an aboveground problem. *Plant Physiol.* 166 689–700. 10.1104/pp.114.245811 25059708 PMC4213098

[B18] Lebeis S. L. Paredes S. H. Lundberg D. S. Breakfield N. Gehring J. Mcdonald M. (2015). Salicylic acid modulates colonization of the root microbiome by specific bacterial taxa. *Science* 349 860–864. 10.1126/science.aaa8764 26184915

[B19] Li X. G. Ding C. F. Hua K. Zhang T. L. Zhang Y. N. Zhao L. (2014). Soil sickness of peanuts is attributable to modifications in soil microbes induced by peanut root exudates rather than to direct allelopathy. *Soil Biol. Biochem.* 78 149–159. 10.1016/j.soilbio.2014.07.019

[B20] Lin S. Huangpu J. J. Chen T. Wu L. K. Zhang Z. Y. Lin W. X. (2015). Analysis of soil microbial community structure and enzyme activities associated with negative effects of *Pseudostellaria heterophylla* consecutive monoculture on yield. *Pak. J. Bot.* 47 761–769.

[B21] Macdonald C. Singh B. (2014). Harnessing plant-microbe interactions for enhancing farm productivity. *Bioengineered* 5 5–9. 10.4161/bioe.25320 23799872 PMC4008467

[B22] Mendes R. Kruijt M. de Bruijn I. Dekkers E. van der Voort M. Schneider J. H. (2011). Deciphering the rhizosphere microbiome for disease-suppressive bacteria. *Science* 332 1097–1100. 10.1126/science.1203980 21551032

[B23] O’Donnell K. Kistler H. C. Cigelnik E. Ploetz R. C. (1998). Multiple evolutionary origins of the fungus causing Panama disease of banana: concordant evidence from nuclear and mitochondrial gene genealogies. *Proc. Natl. Acad. Sci. U.S.A.* 95 2044–2049. 10.1073/pnas.95.5.2044 9482835 PMC19243

[B24] Patten C. L. Glick B. R. (2002). Role of *Pseudomonas putida* indoleacetic acid in development of the host plant root system. *Appl. Environ. Microbiol.* 68 3795–3801. 10.1128/aem.68.8.3795-3801.2002 12147474 PMC124051

[B25] Philippot L. Raaijmakers J. M. Lemanceau P. van der Putten W. H. (2013). Going back to the roots: the microbial ecology of the rhizosphere. *Nat. Rev. Microbiol.* 11 789–799. 10.1038/nrmicro3109 24056930

[B26] Pietro A. D. Madrid M. P. Caracuel Z. Delgado-Jarana J. Roncero M. I. (2003). *Fusarium oxysporum*: exploring the molecular arsenal of a vascular wilt fungus. *Mol. Plant Pathol.* 4 315–325. 10.1046/j.1364-3703.2003.00180.x 20569392

[B27] Punja Z. K. Parker M. (2009). Development of fusarium root and stem rot, a new disease on greenhouse cucumber in British Columbia, caused by *Fusarium oxysporum* f. sp. radicis-cucumerinum. *Can J. Plant Pathol.* 22 349–363. 10.1080/07060660009500453

[B28] Raaijmakers J. M. Weller D. M. (1998). Natural plant protection by 2,4-Diacetylphloroglucinol-Producing *Pseudomonas* spp. in take-all decline soils. *Mol. Plant Microbe Interact.* 11 144–152. 10.1094/mpmi.1998.11.2.144

[B29] Rogers S. O. Bendich A. J. (1985). Extraction of DNA from milligram amounts of fresh, herbarium and mummified plant tissues. *Plant Mol. Biol.* 2 69–76. 10.1007/BF00020088 24306565

[B30] Samson R. Shafik H. Benjama A. Gardan L. (1998). Description of the bacterium causing blight of leek as *Pseudomonas syringae* pv. *porri* (pv. nov.). *Phytopathology* 88 844–850. 10.1094/phyto.1998.88.8.844 18944892

[B31] Tan Y. Ji G. (2010). Bacterial community structure and dominant bacteria in activated sludge from a 70 degrees C ultrasound-enhanced anaerobic reactor for treating carbazole-containing wastewater. *Bioresour. Technol.* 101 174–180. 10.1016/j.biortech.2009.08.044 19733065

[B32] Vujanovic V. Hamel C. H. S. St A. M. (2002). Development of a selective myclobutanil agar (MBA) medium for the isolation of *Fusarium* species from asparagus fields. *Can. J. Microbiol.* 48 841–847. 10.1139/w02-082 12455616

[B33] Weidenhamer J. D. Li M. Allman J. Bergosh R. G. Posner M. (2013). Evidence does not support a role for gallic acid in *Phragmites australis* invasion success. *J. Chem. Ecol.* 39 323–332. 10.1007/s10886-013-0242-y 23328818

[B34] Widmer F. Seidler R. J. Gillevet P. M. Watrud L. S. Giovanni G. D. D. (1998). A highly selective pcr protocol for detecting 16S rRNA genes of the genus *Pseudomonas* (Sensu Stricto) in environmental samples. *Appl. Environ. Microbiol.* 64 2545–2553. 9647828 10.1128/aem.64.7.2545-2553.1998 PMC106424

[B35] Wu H. Wu L. Wang J. Zhu Q. Lin S. Xu J. (2016a). Mixed phenolic acids mediated proliferation of pathogens *Talaromyces helicus* and *Kosakonia sacchari* in continuously monocultured *Radix Pseudostellariae* rhizosphere soil. *Front. Microbiol.* 7:335 10.3389/fmicb.2016.00335 PMC4795122 27014250

[B36] Wu L. K. Chen J. Wu H. M. Wang J. Y. Wu Y. H. Lin S. (2016b). Effects of consecutive monoculture of *Pseudostellaria heterophylla* on soil fungal community as determined by pyrosequencing. *Sci. Rep.* 6:26601 10.1038/srep26601 PMC4877567 27216019

[B37] Wu L. K. Wu H. M. Chen J. Wang J. Y. Lin W. X. (2016c). Microbial community structure and its temporal changes in *Rehmannia glutinosa* rhizospheric soils monocultured for different years. *Eur. J. Soil Biol.* 72 1–5. 10.1016/j.ejsobi.2015.12.002

[B38] Wu L. K. Lin X. M. Lin W. X. (2014). Advances and perspective in research on plant-soil-microbe interactions mediated by root exudates. *Chin. J. Plant Ecol.* 38 298–310. 10.3724/sp.j.1258.2014.00027

[B39] Wu X. Li Z. G. Liu H. J. Chao X. Zhang R. F. Wu H. S. (2015). The effect of long-term continuous cropping of *Black Pepper* on soil bacterial communities as determined by 454 pyrosequencing. *PLOS ONE* 10:e0136946 10.1371/journal.pone.0136946 PMC4552827 26317364

[B40] Yergeau E. Filion M. V. St-Arnaud M. (2005). A PCR-denaturing gradient gel electrophoresis approach to assess *Fusarium* diversity in asparagus. *J. Microbiol. Methods* 60 143–154. 10.1016/j.mimet.2004.09.006 15590089

[B41] Zhang Z. Y. Lin W. X. (2009). Continuous cropping obstacle and allelopathic autotoxicity of medicinal plants. *Chin. J. EcoAgric.* 17 189–196. 10.3724/sp.j.1011.2009.00189

[B42] Zhao W. O. Pang L. Dong N. Yang S. (2015). LC-ESI-MS/MS analysis and pharmacokinetics of heterophyllin B, a cyclic octapeptide from *Pseudostellaria heterophylla* in rat plasma. *Biomed. Chromatogr.* 29 1693–1699. 10.1002/bmc.3481 25967583

[B43] Zhao Y. P. Wu L. K. Chu L. X. Yang Y. Q. Li Z. F. Azeem S. (2015). Interaction of *Pseudostellaria heterophylla* with *Fusarium oxysporum* f. sp. *heterophylla* mediated by its root exudates in a consecutive monoculture system. *Sci. Rep.* 5:8197 10.1038/srep08197 PMC4314652 25645742

[B44] Zhou X. Wu F. (2012). p-Coumaric acid influenced cucumber rhizosphere soil microbial communities and the growth of *Fusarium oxysporum* f. sp. *cucumerinum* Owen. *PLOS ONE* 7:e48288 10.1371/journal.pone.0048288 PMC3484048 23118972

