# Supplementary material for: Assessment of the Diversity of Pseudomonas spp. and Fusarium spp. in Radix pseudostellariae Rhizosphere under Monoculture by Combining DGGE and Quantitative PCR
Source: Front Microbiol. 2017 Sep 15;8:1748. doi: 10.3389/fmicb.2017.01748 (PMC5605650; doi:10.3389/fmicb.2017.01748)
Supplement: Supplementary file 1 [file Data_Sheet_1.pdf]

Supplementary Material

Assessment of the diversity of *Pseudomonas* spp. and *Fusarium* spp. in *Radix pseudostellariae* rhizosphere under monoculture by combining DGGE and quantitative PCR

Jun Chen[1,2], Linkun Wu[1,2], Zhigang Xiao[1,2], Yanhong Wu[1,2], Hongmiao Wu[1,2], Xianjin Qin[2,3], Juanying Wang[1,2], Xiaoya Wei[1,2], Muhammad Umar Khan[1,2], Sheng Lin[1,2], and Wenxiong Lin[1,4]*

1 College of Life Sciences, Fujian Agriculture and Forestry University, Fuzhou, China,

2 Key Laboratory of Crop Ecology and Molecular Physiology, Fujian Agriculture and Forestry University, Fuzhou, China,

3 College of Crop Science, Fujian Agriculture and Forestry University, Fuzhou, China,

4 Fujian Provincial Key Laboratory of Agroecological Processing and Safety Monitoring, Fujian Agriculture and Forestry University, Fuzhou, China

Correspondence: Wenxiong Lin: lwx@fafu.edu.cn

1 Supplementary Figures and Tables

1.1 Supplementary Figures

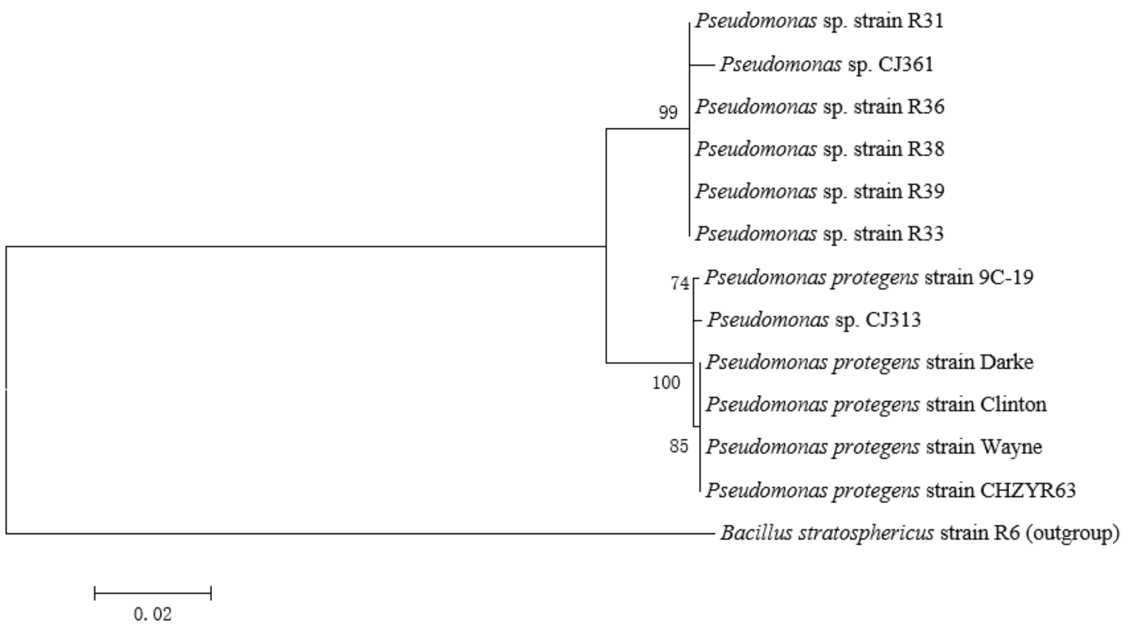

Supplementary Figure S1. Unrooted neighbor-joining tree based on sequence analysis of *Pseudomonas* sp. CJ313 and *Pseudomonas* sp. CJ361 used in this study. Values of the bootstrap analysis (1000 repetitions) are given at the nodes.

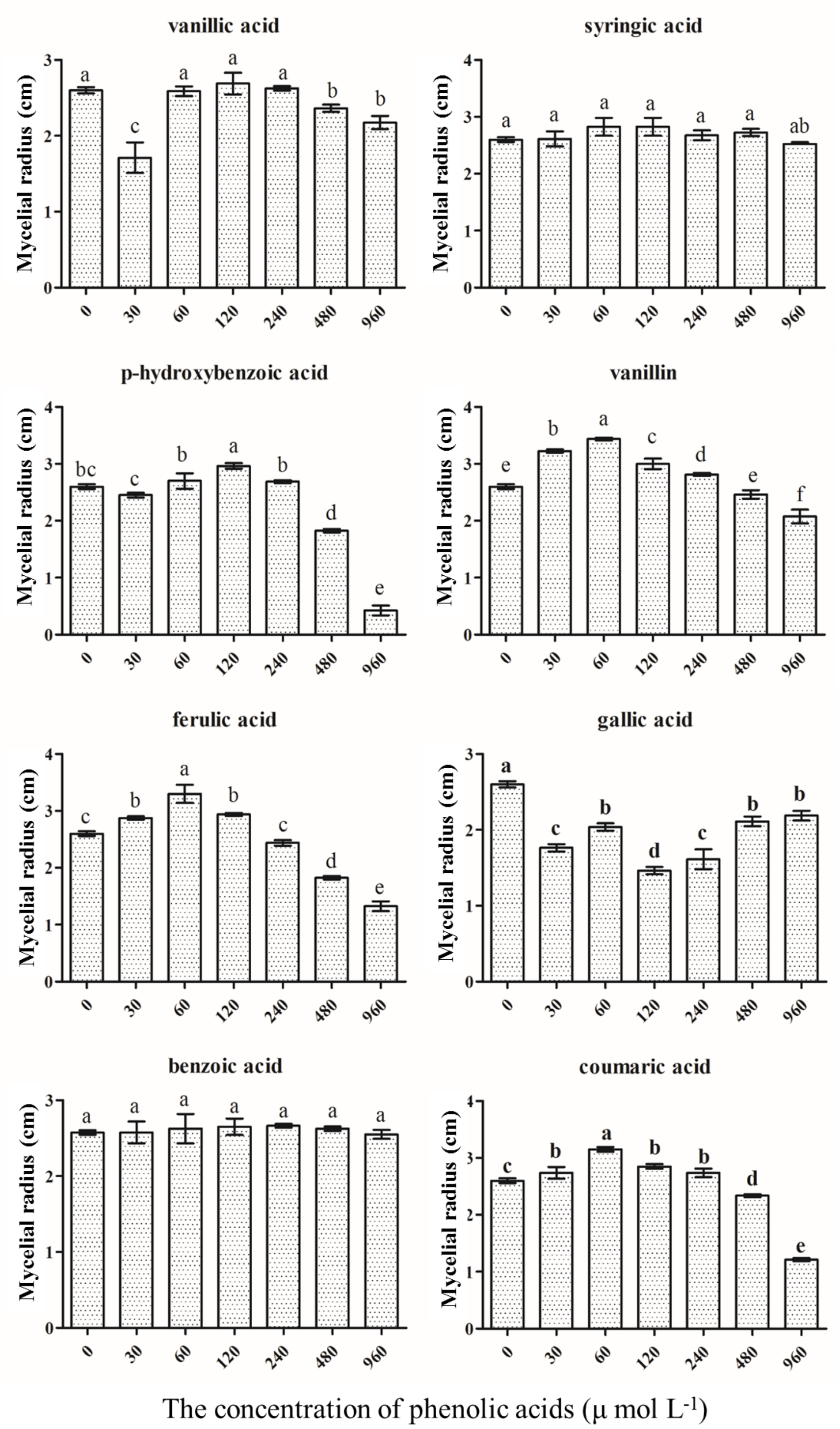

Supplementary Figure S2. Effects of single phenolic acids on the growth of *F. oxysporum*. Data are means ± standard errors (one-way analysis of variance, n = 3).

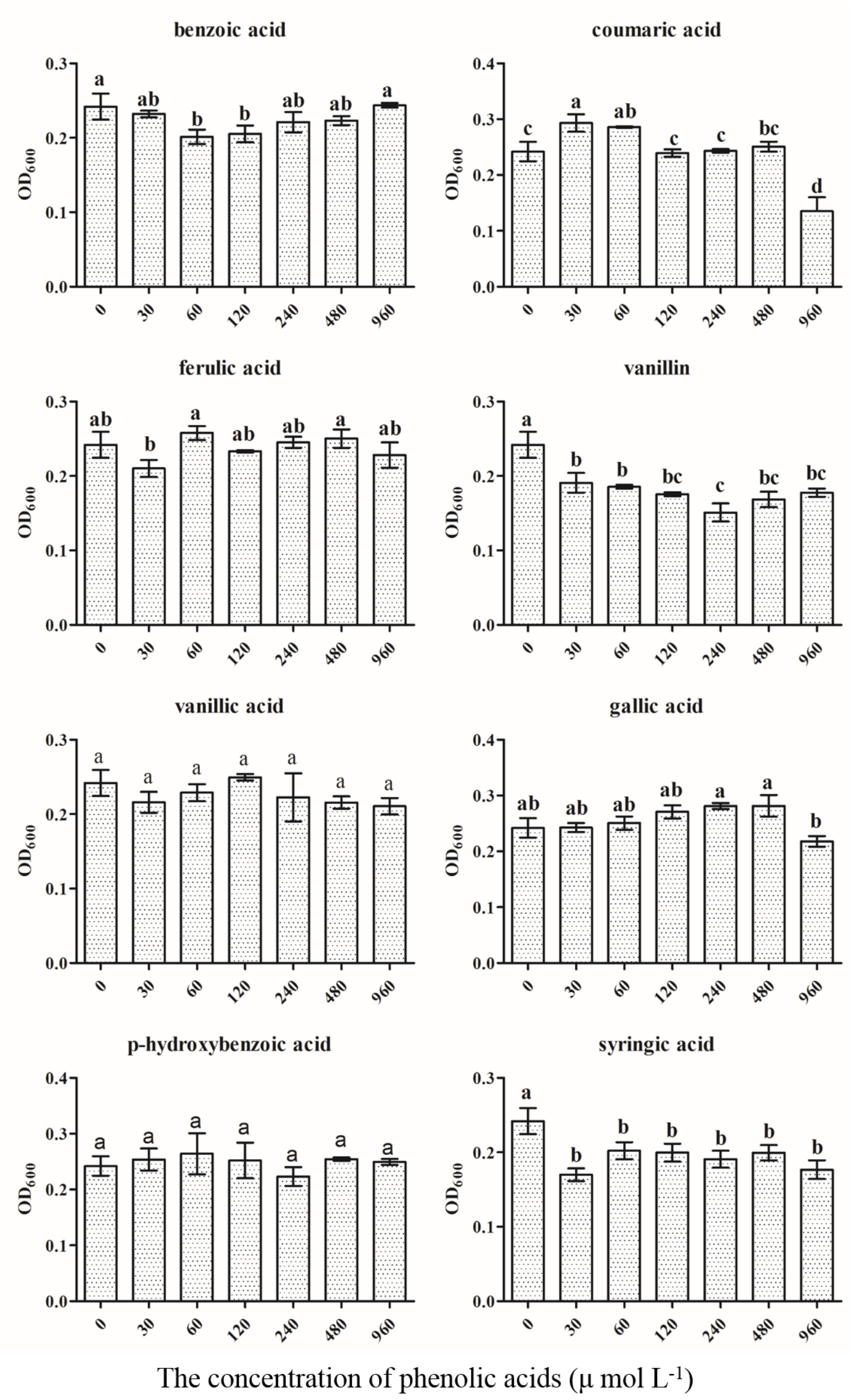

Supplementary Figure S3. Effects of single phenolic acids on the growth of *Pseudomonas* sp. CJ313. Data are means ± standard errors (one-way analysis of variance, n = 4).

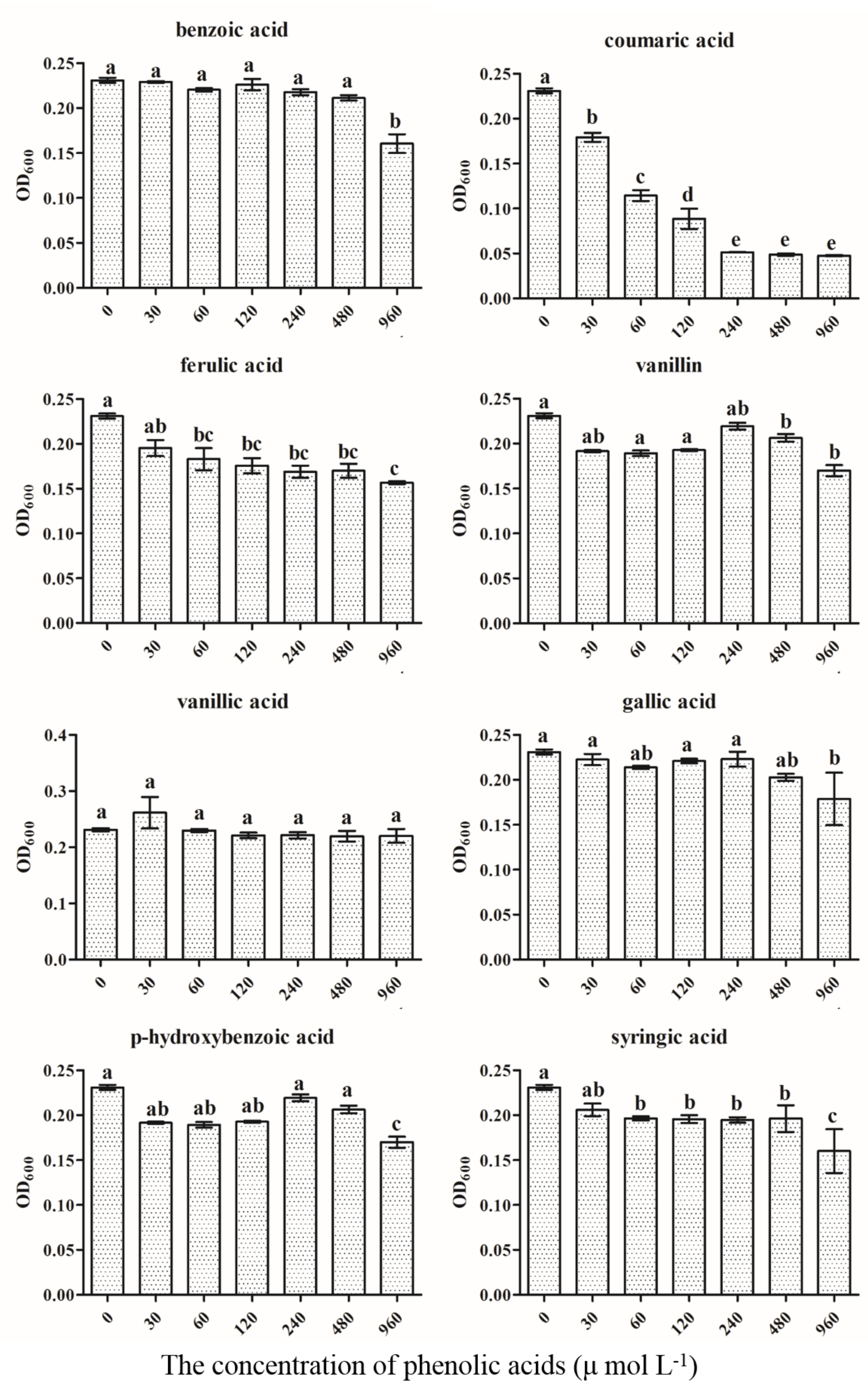

Supplementary Figure S4. Effects of single phenolic acids on the growth of *Pseudomonas* sp. CJ361. Data are means ± standard errors (one-way analysis of variance, n = 4).

1.2 Supplementary Tables

Supplementary Table S1 Taxon-specific primer sets and their thermal conditions for quantitative PCR.

Primer	Sequence (5´- 3´)	Thermal conditions	Reference
Fusarium oxysporum			
ITS1-F	CTTGGTCATTTAGAGGAAGTAA	95℃ for 10min; 95℃ for 50 s, 60.4℃ for 1 min , 72℃ for 1 min, 40 cycles	Lievens et al., 2005
AFP308R	CGAATTAACGCGAGTCCCAA		
***Pseudomonas* sp.**			
Psf	GGTCTGAGAGGATGATCAGT	95℃ for 10min; 95℃ for 1 min, 64℃ for 1 min , 72℃ for 1 min, 40 cycles	Tan and Ji, 2010
Psr	TTAGCTCCACCTCGCGGC		

Supplementary Table S2 Primer sets and their thermal cycling conditions for PCR.

Primer	Sequence (5´- 3´)	Thermal conditions	Reference
**General bacteria**			
27F	AGAGTTTGATCCTGGCTCAG	95℃ for 5min; 95℃ for 1 min, 61℃ for 1 min, 72℃ for 90s, 35 cycles; 72℃ for 10 min	Edwards et al., 1989
1522R	AAGGAGGTGATCCAGCCGCA		
**ITS**			
ITS1F	CTTGGTCATTTAGAGGAAGTAA	95℃ for 5min; 95℃ for 1 min, 51℃ for 45s, 72℃ for 1 min, 35 cycles; 72℃ for 10 min	Gardes and Bruns, 1993; White et al., 1990;
ITS4	TCC TCC GCT TAT TGA TAT GC		

References

Edwards, U., Rogall, T., Blöcker, H., Emde, M., Böttger, and C, E. (1989). Isolation and direct complete nucleotide determination of entire genes. Characterization of a gene coding for 16S ribosomal RNA. *Nucleic Acids Res.* 17, 7843-7853. doi: 10.1093/nar/17.19.7843

Gardes, M., and Bruns, T.D. (1993). ITS primers with enhanced specificity for basidiomycetes-application to the identification of mycorrhizae and rusts. *Mol. Ecol.* 2, 113-118. doi: 10.1111/j.1365-294x.1993.tb00005.x

Lievens, B., Brouwer, M., Vanachter, A.C., Lévesque, C.A., Cammue, B.P., and Thomma, B.P. (2005). Quantitative assessment of phytopathogenic fungi in various substrates using a DNA macroarray. *Environ. Microbiol.* 7, 1698-1710. doi: 10.1111/j.1462-2920.2005.00816.x

Tan, Y., and Ji, G. (2010). Bacterial community structure and dominant bacteria in activated sludge from a 70 degrees C ultrasound-enhanced anaerobic reactor for treating carbazole-containing wastewater. *Bioresour. Technol.* 101, 174-180. doi: 10.1016/j.biortech.2009.08.044

White, T.J., Bruns, T., Lee, S., and Taylor, J. (1990). Amplification and direct sequencing of fungal ribosomal RNA genes for phylogenetics. PCR protocols: a guide to methods and applications. 1, 315-322.
